# Water Quality of a Reservoir and Its Major Tributary Located in East-Central Mexico

**DOI:** 10.3390/ijerph110606119

**Published:** 2014-06-10

**Authors:** Patricia Castilla-Hernández, María del Rocío Torres-Alvarado, José Antonio Herrera-San Luis, Norma Cruz-López

**Affiliations:** 1 Department of the Man and his Environment, Universidad Autónoma Metropolitana-Xochimilco, Calzada del Hueso 1100, Col. Villa Quietud, Coyoacán, C.P. 04960, D.F., Mexico; E-Mails: sanluisantonio.81@gmail.com (J.A.H.-S.L.); norma_cruzlopez@hotmail.com (N.C.-L.); 2 Department of Hydrobiology, Universidad Autónoma Metropolitana-Iztapalapa, Av. San Rafael Atlixco 186, Col. Vicentina, Iztapalapa, C.P. 09340, D.F., Mexico; E-Mail: rta@xanum.uam.mx

**Keywords:** reservoir, dam, Tlaxcala, Atlanga, Zahuapan River, water quality index, WQI

## Abstract

A reservoir with ecological and economic importance and its major tributary, localized in east-central Mexico, were studied. The aim of this work was to know the physicochemical water characteristics of both water bodies and to contrast these by their different uses, and also estimate overall water quality using a Water Quality Index (WQI). Water samples from the reservoir and the tributary were obtained in different climatic seasons. In the tributary, anoxic and hypoxic conditions and high levels of organic matter, orthophosphate, and ammonium showed that this is strongly impacted by wastewater discharges and that the water is not suitable for different uses; independently of the season, the WQI showed “poor” quality (34.4–47.2). In contrast, in the reservoir a better water quality was determined; the WQI in the sampling months ranged from 72.1–76.6 (“good” quality), and spatially, this was from 66.5–79.5 (“fair” and “good” quality).

## 1. Introduction

In Mexico, there are approximately 4,462 man-made reservoirs and water retention berms, and it has been estimated that 150 billion cubic meters of water are stored in these reservoirs [1]. As in other countries [2,3,4,5,6,7], in Mexico dams have been built for different purposes, such as irrigation, to regulate water flow, electric power generation, navigation, fisheries and aquaculture, recreational activities, and for supplying water for public uses [8,9,10].

It is known that deterioration of reservoirs due to anthropogenic factors or natural processes affects the water quality, impairing its future use and also the protection of aquatic life [7,11]; in addition, the characteristics of the water in the tributaries could be an indicator that exerts an impact on these systems or that influences the spatial variations [5,6,12]. Some studies on water quality have been carried out in Mexican reservoirs [10,13,14,15,16]. However, other reservoirs and their tributaries with ecological and economic importance have been poorly studied; with regard to the Atlanga Reservoir, this is the largest and most important of the 16 reservoirs located in the State of Tlaxcala, Mexico, and water from this reservoir is used mostly for supplying agricultural irrigation, mainly in the months of April to August, due to that precipitation in the state is low, with a annual mean of around 700 mm [17]. The Reservoir represents the most important part of Irrigation District 056, denominated Atoyac-Zahuapan. The main crops cultivated in the district are corn, wheat, barley, beans, and oats for fodder, grass, and alfalfa [18].

Furthermore, the Atlanga reservoir is utilized for the fishing of mainly the following six species of carp (*Cyprinus carpio comunis*, *Cyprinus carpio specularis*, *Cyprinus carpio rubrofuscus*, *Hypophtalmichthyus molitrix*, *Carassius auratus*, and *Ctenopharyngodon idellus*) [19], and it supplies water to the Atlangatepec Aquaculture Center, which is devoted to fish reproduction and fry growth, principally of *C. carpio rubrofuscus* and *C. carpio specularis*, for distribution throughout the state*.* The reservoir is also the habitat of a variety of aquatic wildlife [18,20], including endangered species as the axolotl *Ambystoma tigrinum*; additionally, it represents an important site for shelter and nesting of resident birds, and many migratory ones find resources to live here [18]. In 2009, this water body was established as the Ramsar Site and was included in the list of wetlands of international importance [21]. Additionally, there is little information about the water quality of the Zahuapan River, the major tributary of this reservoir [22,23].

On the other hand, and owing to the difficulty in establishing water quality from many parameters, diverse authors have suggested the use of water quality indices (an efficient and simple monitoring tool), because a single number obtained from all of these parameters can indicate the water quality level [10,11,24]; according to this number, water quality can be classified into different ranges as follows: 91–100, excellent; 71–90, good; 51–70, fair; 26–50, poor, and 0–25 very poor [11,25], although depending on the index used the ranges may differ.

Thus, the objective of the present work was to determine the physicochemical water characteristics of the Atlanga Reservoir and its major tributary (Zahuapan River), and to contrast these with the levels established for the purposes for which the reservoir is intended, such as agricultural irrigation, livestock, aquaculture, and protection of aquatic life; also estimate the overall quality of these water bodies by means of a Water Quality Index (WQI). It is expected that information from this work may be useful for decision making regarding the prevention and reduction of tributary pollution, prior to the reservoir reaching lower water quality levels.

## 2. Experimental Section

### 2.1. Study Area

**Figure 1:**
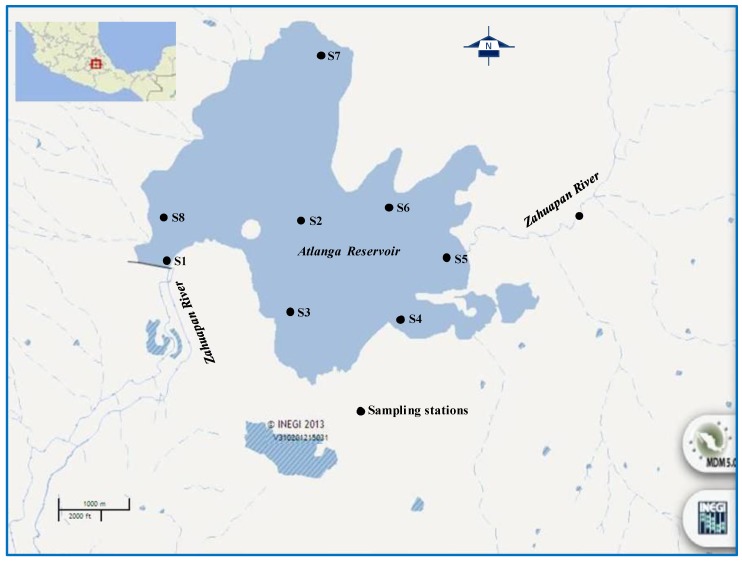
Study area localization and sampling stations in the major tributary (Zahuapan River) and the Atlanga Reservoir. Modified from INEGI [27].

The Atlanga Reservoir is located at an altitude of 2,480 m above sea level and between latitudes 19°32' and 19°34' N and longitudes 98°12' and 98°09' W (Figure 1) in the Atlangatepec Municipality, Tlaxcala State, Mexico. The climate is Cb(w_2_)(w)w''(i')g, which is defined as mild with cool summer and long, sub-humid. The total annual precipitation is of 745.8 mm, December is the driest month and August the wettest, with 7.3 and 135.2 mm, respectively; January presents the highest number of frosts. The winds blow from North to South during the majority of the year [17,26].

The Atlanga Reservoir was inaugurated in 1961; it has a barrier of 24.2 m and includes a superficial area of 1,200 hectares, with a storage capacity of 54.5 million cubic meters and useful storage ranging from 40.9–48.5 million cubic meters [19,28]. The main tributary is the Zahuapan River, a perennial stream; also, the reservoir is fed by two additional, minor intermittent tributaries [18,20]. Average annual inflow volume to the reservoir by the tributaries has been established at 15.11 million cubic meters, while average annual surface rainfall is 6.66 million cubic meters [29]. Forming part of the Atoyac-Zahuapan hydrological system, the Zahuapan River, 82.7 km in length, crosses from the North to the South of the entire state of Tlaxcala and flows into the Atoyac River in Puebla State. Up until 2009, untreated wastewater from the Tlaxco Municipality (39,939 inhabitants) was emptied into the Zahuapan River; in 2010, a wastewater treatment plant was installed outside of this locality.

### 2.2. Water Sampling

At a site on the Zahuapan River (Figure 1), localized at 19°34'13'' N and 98°09'6.59'' W, upstream of the Atlanga Reservoir, during the months of March, May, July, and September 2009, water samples were collected every 2 h for a period of 12 h (7:30 a.m. to 7:30 p.m.) at a depth of 20 cm. Water samples were collected with a Van Dorn bottle and preservation for their later analysis was carried out according to APHA-AWWA-WEF [30]. Dissolved oxygen (DO), temperature (*T*), transparency, and pH were determined *in situ.*

In the reservoir and during the same year, six samplings, corresponding to January, March, May, July, September, and November, were conducted. The samples were collected from the dam at eight sampling stations denominated as follows: Barrier (S1); Center (S2); Agricultural (S3); Airport Jetty (S4); Zahuapan Discharge (S5); San Luis (S6); Santa Clara (S7), and Fuerte Apache (S8), as depicted in Figure 1. The samples were collected at a depth of 50 cm and were conserved as previously mentioned. Depth, DO, *T*, transparency, and pH were analyzed *in situ*. 

### 2.3. Sample Analysis

Samples were analyzed for several parameters. Total suspended solids (TSS) were calculated for weight differences after drying at 105 °C (method 2540 D); organic matter (OM), such as chemical oxygen demand, utilized the closed reflux method with potassium dichromate (K_2_Cr_2_O_7_) as oxidant (method 5220 D); DO by iodometric method (4500-O B); nitrite nitrogen (NO_2_^−^-N) by Griess reaction (method 4500-NO_2_-B), and phosphorus of orthophosphate (PO_4_^3−^-P), by ascorbic acid methods (4500-P E) as reported by APHA-AWWA-WEF [30]. On the other hand, nitrate nitrogen (NO_3_^−^-N) was analyzed by the Hach method (cadmium reduction), ammonium nitrogen (NH_4_^+^-N) with a selective electrode (Orion, Beverly, MA, USA), and hardness (calcium carbonate (CaCO_3_)) as indicated in Mexican Norm [31]. For colorimetric techniques, the samples were previously filtered onto Whatman membranes (0.45-µm pore size, 47 mm in diameter), and a Genesys 20 spectrophotometer (Madison, WI, USA) was used. For measuring transparency we employed a Secchi disk, a mercury thermometer for *T*, and for pH, a potentiometer (Conductronic, Puebla, Mexico) previously calibrated with 4.0 and 7.0 buffers; while reservoir depth was determined with a portable sounder (Speedtech Instrument, Great Falls, VA, USA).

### 2.4. Data Analysis

For the individual parameters, means and standard deviation (SD) were obtained. The means were compared by one-way Analysis of variance (ANOVA); when significant *p* values were obtained, a multiple comparison Tukey test was performed. Student *t* test comparisons were utilized for only two of the means. For all parameters (9 for the tributary and 10 for the reservoir), the WQI reported by Pesce and Wunderlin [24], and widely used by the evaluation of overall water quality of different water bodies [11,32], was calculated using equation (1):

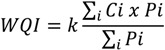
(1)


The parameters normalization and assigned weights for the WQI are according to values proposed for aquatic life preservation [24]; the water quality for this purpose may be defined as the required quality to maintain the interactions and interrelationships of living organisms according to the natural balance of the aquatic ecosystems [33].

To calculate the WQI, the concentration of each parameter (except transparency) was normalized (*Ci*) by assigning values ranging from 0–100% as was previously reported [24] (Table 1), where 100 represents maximal quality, and zero the lowest; for transparency, normalization was carried out according to water quality levels reported by aquaculture [33], where *Ci* 100 was assigned from twice 50 cm, the upper value established for carp, and of 70 to 50 *Ci* values, corresponding to 50–30 cm, the range established for this fish. Additionally, each parameter was assigned a relative weight (*Pi*) within a range from 1–4, where 1 is assigned to the parameter least important for aquatic life preservation, and 4, the most important; in this case, number 4 was assigned to DO, number 3 to NH_4_^+^-N and to OM, number 2 to NO_2_^−^, NO_3_^−^ and transparency, and number 1 to hardness, pH, PO_4_^3−^, and to *T*. Finally, for the *k* constant, which represents the visual appearance concerning the contamination of the water body and that could be evaluated by an individual without training in environmental knowledge, the value assigned was 1; when *k* receives this value, the index is known as the objective WQI [24].

**Table 1:** Parameters used in the calculation of the WQI, relative weights assigned (*Pi*) to each parameter, and *Ci* values utilized in the normalization.

Parameter	*Pi*	*Ci*										
		100	90	80	70	60	50	40	30	20	10	0
**DO**	4	≥7.5	>7.0	>6.5	>6.0	>5.0	>4.0	>3.5	>3.0	>2.0	≥1.0	<1.0
**NH_4_^+^-N**	3	<0.01	<0.05	<0.10	<0.20	<0.30	<0.40	<0.50	<0.75	<1.00	≤1.25	>1.25
**OM**	3	<5	<10	<20	<30	<40	<50	<60	<80	<100	≤150	>150
**NO_2_^−^**	2	<0.005	<0.01	<0.03	<0.05	<0.10	<0.15	<0.20	<0.25	<0.50	≤1.00	>1.00
**NO_3_^−^**	2	<0.5	<2.0	<4.0	<6.0	<8.0	<10.0	<15.0	<20.0	<50.0	≤100.0	>100.0
**Transparency**	2	>100	>70	>60	>50	>40	>30	>25	>20	>15	≥10	<10
**Hardness**	1	<25	<100	<200	<300	<400	<500	<600	<800	<1,000	≤1,500	>1,500
**pH**	1	7	7–8	7–8.5	7–9	6.5–7	6–9.5	5–10	4–11	3–12	2–13	1–14
**PO_4_^3−^**	1	<0.16	<1.60	<3.20	<6.40	<9.60	<16.0	<32.0	<64.0	<96.0	≤160.0	>160.0
***T***	1	21/16	22/15	24/14	26/12	28/10	30/5	32/0	36/−2	40/−4	45/−6	>45/<−6

Notes: Values in mg/L; pH in pH units; T in °C; transparency in cm. Transparency was not used to calculate the WQI for the tributary and for the WQI presented in Figure 5a and Figure 5b.

## 3. Results and Discussion

### 3.1. Tributary

**Figure 2:**
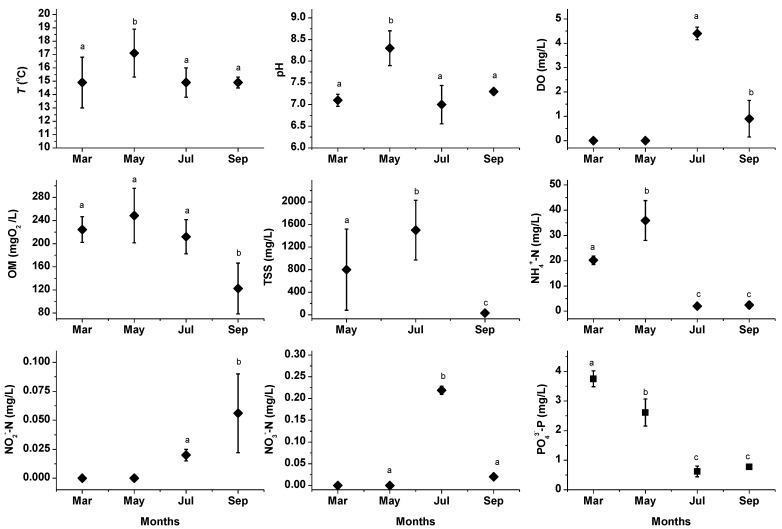
Physical and chemical parameters in the tributary. The Standard deviation (SD) represents the dispersion among scheduled samplings; dissimilar lowercase letters on the SD bars indicate significant differences in Tukey multiple comparison or Student *t* tests.

The results are presented in Figure 2. *T* varied considerably among the sampling schedules of the first 3 months and analysis of mean multiple comparison shows that the highest was in May (17.1 ± 1.8 °C) (*F* = 4.6265; *p* < 0.05; Tukey, *p* < 0.05). In March, July, and September, this parameter was 14.9 ± 1.9, 14.9 ± 1.1, and 14.9 ± 0.4 °C, respectively, despite the seasonal difference. pH had a similar pattern with a increase in May, with a value of 8.3 ± 0.40 (*F* = 26.636, *p* < 0.05; Tukey, *p* < 0.05), and in March, July, and September, this remained nearly neutral (7.1 ± 0.14, 7.0 ± 0.44, and 7.3 ± 0.07, respectively). Anoxic conditions in March and May were determined; in July, OD was 4.4 ± 0.26 mg/L and no significant differences were observed across the sampling schedules. In September, hypoxic conditions were determined in the first 6 h, with an average of 0.9 ± 0.75 mg/L. Differences were found between these months (*t* = −11.408; *p* < 0.05 ), although both belonged to the rainy period.

The OM concentration presented high variation between the schedules and exhibited the highest levels from March to July (means > 200 mgO_2_/L); even when DO was present in July, this registered similar concentrations to those of the previous months (Tukey, *p* > 0.05). In September, the OM concentration decreased (122.2 ± 44.0 mgO_2_/L), probably due to an increase in river flow. Otherwise, in May, TSS concentration was 802 ± 726 mg/L, and in July, this concentration was duplicated (1,529 ± 536 mg/L). In both months, the high content may be caused by the beginning of the rainy season and the heavy rains, which drag the accumulated solids into the riverbed and onto the surrounding land. The lowest level of solids was established in September with 29.9 ± 22.8 mg/L, and means found in the months were statistically different (*F* = 14.486; *p* < 0.05; Tukey, *p* < 0.05). Additionally, inorganic solids predominated (≈80%) at all times.

*T* and pH fell within the permissible limits according to Protection of Aquatic Life (PAL) and Agricultural Irrigation (AI), as established by the Mexican Norm [33]. However, in three of the four sampling months, DO was found under critical conditions with respect to the minimal allowable limit according to PAL, that is, 5.0 mg/L [33], coinciding with OM concentrations, which can be classified into polluted and heavily polluted, indicating a strong impact of wastewater discharges [1]. It is known that medium- and small-sized rivers, such as the Zahuapan, are more affected by anthropogenic pollution than large rivers [34]. DO levels in the Zahuapan River were comparable with those found during other years [22] and with those of other heavily polluted rivers, such as the Lerma River, in which DO has been found at <1 mg/L [16]. OM was similar to those found in the Atoyac River, which is impacted by the Zahuapan River and by wastewater discharges [35]. Also, the TSS levels found in May and July were not appropriated for AI, because <50 mg/L are established for this use [33], or water containing >75 and ≤150 mg/L is restricted for this use [1]. 

With regard to the inorganic nitrogen analyzed, NH_4_^+^-N was the predominant form and the means were significantly different (*F* = 107.511; *p* < 0.05); in March, we found 20.2 ± 1.7 mg/L, while in May, this level increased in the scheduled samplings to 49.5 mg/L, with a mean of 35.9 ± 7.9 mg/L; Tukey (*p* < 0.05) showed differences. In July and September, and apparently due to the rainy season (Figure 4b), the concentration decreased to 2.0 ± 0.8 and 2.4 ± 1.3 mg/L, respectively, and both were similar (Tukey, *p* > 0.05). The existence of NH_4_^+^ in water is an indicator of direct pollution [12], and it is known that this can be toxic to aquatic life; in this respect, in all sampling months the ammonium concentrations exceeded the maximum permissible limit of 0.06 mg NH_4_^+^-N/L recommended [33], surpassing the concentrations of around 2.0–5.8 mg/L reported by Barceló-Quintal *et al*. [16].

Oxidized forms, such as NO_2_^−^-N, were not present during the first two months, while in July and September, these were 0.02 and 0.056 mg/L, respectively, and differences were found (*t* = 2.779; *p* < 0.05). NO_3_^−^-N was not detected in March; in May and September, it was registered at low levels (<0.1 mg/L) due to the lack of or low DO concentration, which did not favor NH_4_^+^-N oxidation, while in July this reached 0.22 mg/L. NO_2_^−^-N and NO_3_^−^-N levels were lower than 10 and 90 mg/L, maximum limits established for livestock use, respectively [33].

Phosphorus is a nutrient that can promote the development of species that accelerates the eutrophication process. In this tributary, PO_4_^3−^-P concentrations were highest in March (3.7 ± 0.27 mg/L), followed by May with 2.6 ± 0.46 mg/L (*F* = 211.814, *p* < 0.05; Tukey, *p* < 0.05), and these decreased in the following months to minimum values (0.62 ± 0.18 and 0.78 ± 0.05 mg/L), during which the concentrations were not statistically different (Tukey, *p >* 0.05). All of the concentrations determined were >0.1 mg/L of phosphates, the maximal level permitted to control the accelerated eutrophication, in rivers and streams [33]; the high levels of PO_4_^3−^-P may be largely associated with wastewater discharges containing detergents [12], although during the rainy season, fertilizer runoff from adjacent agricultural areas can contribute to these.

Hardness was higher in May and in September, reaching 326.4 ± 47.9 and 288.0 ± 6.9 mg/L, respectively (*F* = 21.174; *p* < 0.05; Tukey, *p >* 0.05), and decreased in July (225.5 ± 15.2 mg/L), exhibiting significant differences with respect to the remaining months (data not shown in Figure 2); by the levels found of hardness, the water was classified as very hard [36].

### 3.2. Reservoir

**Figure 3:**
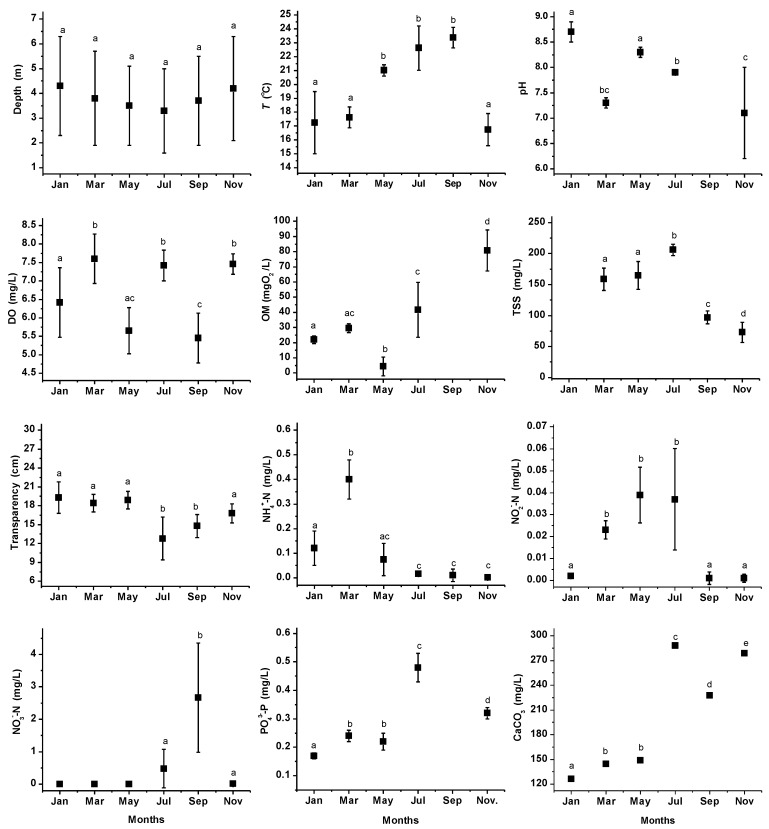
Physical and chemical parameters obtained for the reservoir during the 6 sampling months. Standard deviation (SD) bars correspond to the dates registered for the eight sampling stations within the reservoir. Dissimilar lowercase letters on the SD bars indicate significant differences in Tukey multiple comparison tests.

Results in the different sampling months are presented in Figure 3, while those corresponding to the sampling stations are presented in Table 2. A decrease in depth was observed from January to May (4.3 ± 2.0 to 3.5 ± 1.6 m) and an increase was observed again from July to November (3.3 ± 1.7 to 4.2 ± 2.1 m); however, due to the heterogenic conditions at the sampling stations, significant differences were not found among the months (*F* = 0.347; *p* > 0.05). Among sampling stations, Stations S1 and S2 had the greatest depth (around 6.0 m), and Stations S4 to S6 exhibited shallow conditions, although the S5 sampling station (Zahuapan Discharge) presented the lowest level (0.9 ± 0.3 m); this could be due to hauling solids through the river and subsequent release into the reservoir, for example, 1,529 ± 536 mgTSS/L (Figure 2), causing accumulation at this site.

Water *T* presented significant differences (*F* = 40.01; *p* < 0.05; Tukey, *p* < 0.05) and was higher from May to September (21.0 ± 0.4 to 23.4 ± 0.7 °C), corresponding to the warmer season, and was lower in the remaining months (16.8 ± 1.2 to 17.6 ± 0.7 °C) according to the seasonal profile, as exhibited at other reservoirs [7]. At the sampling stations, *T* ranged from 19.1 ± 3.2 to 20.6 ± 2.3 °C. The levels found for this parameter are suitable for the different water uses [33].

Throughout all of the months, the pH presented significant variation (*F* = 19.064; *p* < 0.05); January and May demonstrated alkaline conditions with levels ≥8.3 (Tukey, *p* < 0.05), followed by March and July, which ranged from 7.3 ± 0.1 to 7.9 ± 0.04. In November, a wide variation was determined due to the alkaline tendency at six sampling stations and to the acidic conditions found at stations S4 and S5, which reached minimal values of 6.0. Chapman and Kimstach [37] mention that in the majority of natural water, the pH is found to be 6.0–8.5, but higher levels can be observed in eutrophic waters or low levels can occur in waters with high organic content. The pH levels were appropriate for AI, with a range of 4.5 to 9.0 established for this purpose; but in January and November, this parameter was slightly outside of the range reported to carp aquaculture (7.0−8.5) [33].

Regarding DO concentration, this presented monthly significant differences (*F* = 18.282; *p* < 0.05). In March, July, and November, the reservoir exhibited highest levels (7.4 ± 0.4 to 7.6 ± 0.7 mg/L) and these were similar (Tukey, *p* > 0.05), while in January, concentration diminished and there was high variation among stations (6.4 ± 0.9 mg/L). In May and September, levels were lower (5.7 ± 0.6 and 5.5 ± 0.7 mg/L, respectively), and at this time, stations S4 and S5 registered levels <5.0 mg/L, not suitable for PAL and carp aquaculture [33]. At sampling stations, there were mean concentrations ranging from 6.2 ± 1.2 to 6.9 ± 1.4 mg/L. In all sampling months, DO levels were suitable for PAL and carp aquaculture, except for September, during which the concentration was slightly below the permissible limit. 

Significant differences were found for OM (*F* = 57.982; *p* < 0.05), and January and March achieved acceptable concentrations (22.0 ± 2.6 and 29.6 ± 2.9 mgO_2_/L, respectively). In May, these levels diminished to 4.4 ± 6.2 mgO_2_/L and exhibited good quality, while in July and November, the levels increased to 41.7 ± 18.1 and 80.7 ± 13.5 mgO_2_/L, respectively, and showed a significant difference (Tukey, *p* < 0.05), reaching levels of polluted water [1]. At the sampling stations, this parameter ranged from 28.9 ± 24.9 to 40.4 ± 34.8 mgO_2_/L. A significant diminution with respect to that found in the tributary was observed at Station S5, probably due to the partial degradation of organic matter; consequently, a negative effect on DO and pH at this station was observed.

**Table 2:** Means of the physical and chemical parameters at the dam sampling stations are presented. Standard deviation (SD) (values within parentheses) illustrates the dispersion found at each station during the 6 sampling months.

Parameter	Sampling Stations							
	S1	S2	S3	S4	S5	S6	S7	S8
**Depth (m)**	6.0	6.2	3.6	2.5	0.9	2.5	4.5	4.2
	(0.7)	(0.5)	(0.8)	(0.5)	(0.3)	(0.5)	(0.3)	(1.2)
***T* (°C)**	19.2	19.1	19.3	20.3	20.6	20.0	19.8	19.9
	(2.9)	(3.2)	(2.5)	(3.3)	(2.3)	(3.8)	(3.3)	(3.7)
**pH**	7.9	8.0	8.0	7.5	7.6	7.8	8.0	8.1
	(0.8)	(0.5)	(0.6)	(1.2)	(1.0)	(0.9)	(0.5)	(0.5)
**DO (mg/L)**	6.8	6.8	6.9	6.2	6.5	6.9	6.4	6.9
	(0.8)	(0.9)	(0.8)	(1.2)	(1.6)	(1.4)	(1.2)	(0.9)
**OM (mgO_2_/L)**	33.7	36.0	38.7	37.6	34.1	28.9	36.0	40.4
	(23.0)	(34.0)	(32.9)	(30.6)	(30.3)	(24.9)	(30.6)	(34.8)
**TSS (mg/L)**	134.4	144.3	147.1	151.3	144.6	135.0	126.8	135.5
	(51.8)	(44.0)	(46.6)	(65.5)	(56.8)	(67.4)	(56.1)	(54.8)
**Transparency (cm)**	17.3	15.7	18.0	16.8	15.6	15.7	17.2	18.2
	(1.2)	(3.4)	(2.2)	(2.3)	(4.1)	(3.3)	(4.3)	(3.7)
**NH_4_^+^-N (mg/L)**	0.13	0.09	0.09	0.07	0.12	0.11	0.09	0.13
	(0.15)	(0.16)	(0.12)	(0.12)	(0.19)	(0.16)	(0.16)	(0.20)
**NO_2_^−^-N (mg/L)**	0.013	0.011	0.023	0.016	0.021	0.018	0.019	0.018
	(0.021)	(0.015)	(0.024)	(0.018)	(0.018)	(0.018)	(0.021)	(0.021)
**NO_3_^−^-N (mg/L)**	0.32	0.14	0.47	0.61	0.36	0.79	0.65	0.88
	(0.5)	(0.2)	(1.2)	(1.5)	(0.07)	(1.7)	(1.6)	(1.8)
**PO_4_^3−^-P (mg/L)**	0.28	0.28	0.28	0.25	0.29	0.29	0.31	0.31
	(0.12)	(0.13)	(0.13)	(0.10)	(0.10)	(0.12)	(0.14)	(0.15)
**CaCO_3_ (mg/L)**	204.0	202.2	203.8	204.5	202.7	205.0	199.6	197.0
	(69.5)	(74.3)	(72.6)	(70.6)	(73.6)	(78.1)	(70.4)	(66.4)

TSS content showed similar concentrations in March and May (160 mg/L) (Tukey, *p* < 0.05); in July, an increase was detected (206.1 ± 9.2 mg/L). During these months, the levels of this parameter were found among those of polluted water [1]; TSS diminished significantly in September (96.9 ± 10.4 mg/L) and November (73.0 ± 16.3 mg/L), reaching levels of acceptable and good water quality [1]. At the sampling stations, TSS concentrations were found to range from 126.8 ± 56.1 to 151.3 ± 65.5 mg/L, and the inorganic fraction was high (85.6%). The levels were >50 mg/L, limit permitted for AI [33]. High levels of TSS are caused by soil type and deforestation; also, winds promote these to remain suspended, in contrast to other lakes or reservoirs, in which during the dry season these exhibit a tendency toward sedimentation [9]. It was reported that concentrations of suspended solids of 40–200 mg/L can reduce primary production from 13 to 50% [38]. Additionally, although it is known than cyprinid fish such as carp are more tolerant to high levels of solids [38], in this reservoir it appears that the latter have a negative effect on the natural reproduction of carp, and fisheries are dependent on artificial reseeding [19]. To reduce the effect of the solids, the water should be previously treated prior to its use at the local Aquaculture Center.

Transparency was similar from January to May (19.3 ± 2.5 to 18.9 ± 1.4 cm; Tukey; *p >* 0.05); in July and September, this decreased to 12.8 ± 3.4 and 14.8 ± 1.8 cm, respectively. Sampling stations exhibited levels from 15.6 ± 4.1 to 18.2 ± 3.7 cm, demonstrating high variation. Transparency levels established for aquaculture (specifically carp) range from 30–50 cm [33]. The levels found for transparency (<22.0 cm) corresponded to very turbid water and these were lower than the 0.7–2.5 m reported for other reservoirs [5,6]. It has been reported that turbidity does not necessarily correspond to the level of suspended solids, due to that this is excessively influenced by the presence of dissolved mineral substances, dissolved humic substances, and phytoplankton [38]; however, at this reservoir, the high inorganic fraction indicates a predominance of suspended solids.

In terms of NH_4_^+^-N, the concentration was highest in March, reaching 0.40 ± 0.08 mg/L (*F* = 71.424; *p* < 0.05; Tukey, *p* < 0.05), while in January and May, we found concentrations of 0.12 ± 0.07 and 0.07 ± 0.06 mg/L, respectively, exceeding the limit permitted for use in PAL [33]. From July to November, this parameter presented good levels for different uses (<0.02 mg/L). Sampling stations presented levels ranging from 0.07 ± 0.12 to 0.13 ± 0.20 mg/L. 

In January, September, and November, NO_2_^−^-N was present at low and similar concentrations (<0.003 mg/L; Tukey, *p* < 0.05), with a significant increase between March and July (0.023 ± 0.004 to 0.037 ± 0.02 mg/L); at sampling stations, concentrations ranged from 0.011 ± 0.015 to 0.023 ± 0.024 mg/L. From January to May, NO_3_^−^-N was not found; in July and November, it was detected (0.48 ± 0.6 and 0.02 ± 0.03 mg/L) and there was not a significant difference (Tukey; *p* > 0.05), but a significant increase was found in September (2.7 ± 1.7 mg/L). At the sampling stations, the concentrations of this parameter ranged from 0.14 ± 0.2 to 0.88 ± 1.8 mg/L, and Stations S1 and S2 exhibited the lowest concentrations. To both parameters, all of the levels were optimal for livestock [33].

The PO_4_^3−^-P concentration exhibited a significant difference among the months (*F* = 159.42; *p* < 0.05), with January presenting the lowest concentration (0.17 ± 0.01 mg/L), followed by March and May, which had similar levels (0.24 ± 0.02 and 0.22 ± 0.03 mg/L, respectively; Tukey, *p* > 0.05). An increase was observed in July, reaching 0.48 ± 0.05 mg/L. In November, the level decreased to 0.32 ± 0.02 mg/L. Among sampling stations, homogeneous concentrations was presented and ranged from 0.25 ± 0.10 to 0.31 ± 0.15 mg/L. PO_4_^3−^-P levels in the sampled months and at the stations were >0.025 mgP/L, the maximal limit established for dams [33]. This reservoir presented levels of this nutrient that were 10 times higher than those of other reservoirs that receive point sources, such as municipal effluents, and non-point sources including atmospheric deposition, and agricultural runoff [7].

Water hardness was different among the sampling months (*F* = 159.42; *p* < 0.05); 126.2 ± 5.3 mg/L were found in January, and in March and May, the concentrations showed no significant difference (144.3 ± 2.4 and 149.1 ± 2.7 mg/L; Tukey, *p* > 0.05). From July to November, highest concentrations were reached (≥224.0 and ≤296.0 mg/L, respectively), and there were differences among these (Tukey, *p* < 0.05). Sampling stations presented concentrations ranging from 197.0 ± 66.4 to 205.0 ± 66.4 mg/L. The water was classified as hard and very hard [36], but the levels found were adequate for carp aquaculture (<300 mg/L) [33].

### 3.3. Water Quality Index

**Figure 4:**
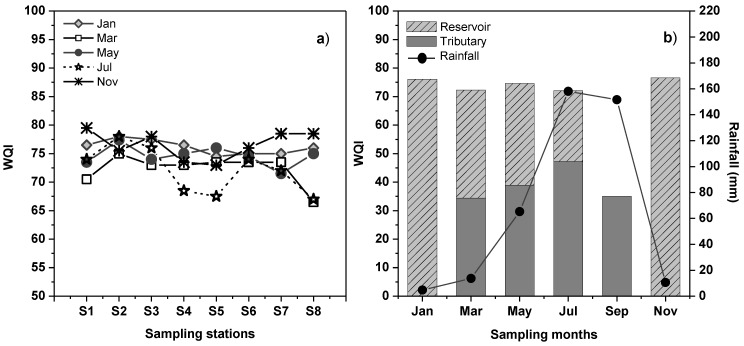
(**a**) Water quality index (WQI) obtained for sampling stations of the reservoir. (**b**) Tributary and reservoir comparison.

In the reservoir, the WQI presented low variation, which ranged from 66.5 (Station S8, March) to 79.5 (Station S1, November), as depicted in Figure 4a. In general, highest WQI levels were found at Station S2 at all times (76.8 ± 1.4), corresponding to the reservoir’s Center and Transition zone, this quality may be attributed to the low levels and scantly variation of NO_2_^−^-N and NO_3_^−^-N found in this site. The stations S3 and S1 reached a WQI of 75.7 ± 2.2 and 74.8 ± 3.4, respectively, corresponding to the Agricultural and Barrier of the reservoir; thus, the water delivered downstream was ranged from 70.5–79.5.

Lowest WQI value was found for Station S5, the site of the discharge of the tributary (72.9 ± 3.2) and that exhibited typical characteristics of the riverine zone in the reservoirs, because this zone receives a high input of nutrients [39]; this could explain the quality found in this site. The second lowest WQI value was achieved by Station S4 (73.3 ± 3.0), a site very near Station S5; additionally, Station S4 is where boats leave most frequently to perform fishing activities. Station S8 presented a heterogeneous level with 72.6 ± 5.5; in March and in July, levels of 66.5 and 67 were found; this site is used for tourist activities on weekends, although these activities are extremely low compared with those at other Mexican tourist sites. Finally, all sample stations were classified with “good” water quality, except Stations S4 and S5 (in the month of July) and Station S8 (in the months of March and July), during which levels fell to a water quality of “fair”.

In the reservoir during the months studied, “good” water quality was determined (72.1–76.6) (Figure 4b), and unlike the pattern observed in single environmental variables that was associated with the dry or the rainy season, the WQI was not related with weather conditions; for example, very dry months (such as March) resulted as similar to months with intense rainfall (July). 

In the tributary, the WQI reached values from 34.4–38.9, corresponding to March, May, and September; in July, a slight increase was observed (47.2), probably due to the increase in DO and the increase of rainfall, which acted as a dilution factor, although all calculated levels fell within the range of “poor” water quality (Figure 4b). These levels showed the impact that wastewater exerts on the river, and although in the rainy season there is an increase in the flow and DO concentration and a decrease was found of nutrients such as NH_4_^+^-N or PO_4_^3−^-P, the water quality did not improve significantly.

However, when the water from the tributary was released into the reservoir, an important increase in the WQI was observed; the “poor” quality of the tributary was reverted in the reservoir, probably due to the following different factors: 1) the contribution of primary producers (by nutrient utilization and oxygen production), among these phytoplankton, although aquatic vegetation, such as the tule (*Scirpus lacustris L.*), could be an especially strong contributor because there is a strip on the edge of the reservoir and observations in the study area have revealed a higher abundance of this plant in recent years. Regardless of this, this plant appears to entertain a duality, because if it continues growing so excessively, it can have adverse effects on the site in terms of gaining ground and contributing to eutrophication. Notwithstanding this, the following have been observed: (1) the tule has served to shelter birds and their nests, and it is the habitat of other aquatic organisms [18], and (2) the frequency and dominance of the north winds that together induce the surface water mixing, promoting oxygen diffusion and aiding in biogeochemical recycling as, for example, when OM and NH_4_^+^ levels are high and additional oxygen is necessary for their oxidation into CO_2_ and NO_3_^─^, respectively [40]; as well as when oxygen consumption by respiration is greater than production from primary producers, and (3) the long residence time (685.7–813.6 days), which facilitates the loss of carbon, phosphorus, and nitrogen by sedimentation, mainly in the riverine zone of the reservoirs [41]; conversely, the remaining available nutrients have a much greater opportunity for utilization by the primary producer, promoting an increase in eutrophication.

**Figure 5:**
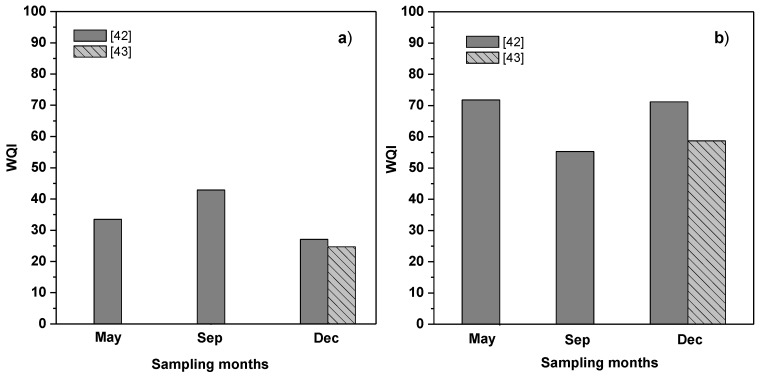
Water quality index (WQI) from dates of the year 2011 [42,43]. (**a**) Tributary and (**b**) Reservoir (Station 1).

Finally, in order to know the evolution of water quality in the reservoir and its tributary, from parameters reported by two studies [42,43], in some months of the year 2011 and at sites corresponding to the tributary upstream of the Atlanga Reservoir and the reservoir’s barrier, WQI was calculated, in the tributary ranged from 24.7–42.9 (Figure 5a), while in the reservoir, this ranged from 55.3–71.2 (Figure 5b). In relation to this, it was expected that in later years to 2009, the water quality would be of similar or of better quality, because a wastewater treatment plant was installed outside of the locality that is the major pollution source in this zone, nonetheless, this was not so; but, other studies with more frequent samplings and longer periods are necessary to come to conclusions about these. 

## 4. Conclusions

In the tributary, anoxic and hypoxic conditions and high levels of OM, PO_4_^3−^-P, and NH_4_^+^-N showed that the tributary was strongly impacted by wastewater discharge; due to the levels found in several parameters, the water is not suitable for PAL and in some time for AI uses, and the WQI reported “poor” quality (34.4–47.2), independent of the season. In the reservoir, the physicochemical conditions showed better levels, due to the contribution of biological and physical factors, and the WQI during the sampling months ranged from 72.1–76.6 (“good” quality), while spatially, this ranged from 66.5–79.5 (“fair” and “good” quality), and 10% of these values were found at <70%. Due to all of the latter, and although the reservoir presented best water quality, it is necessary to pay attention to the tributary (in order to reduce and avoid pollution), before the reservoir reaches lower water quality levels and exerts a negative impact on the activities of protection of the aquatic life, irrigation, fisheries, and aquaculture, practiced at the reservoir. 
